# Serum GGT/ALT ratio predicts vascular invasion in HBV-related HCC

**DOI:** 10.1186/s12935-021-02214-1

**Published:** 2021-09-28

**Authors:** Zhifeng Zhao, Yiming Zhu, Xiaochun Ni, Jiayun Lin, Hongjie Li, Lei Zheng, Chihao Zhang, Xiaoliang Qi, Haizhong Huo, Xiaolou Lou, Qiang Fan, Yongyang Bao, Meng Luo

**Affiliations:** 1 Department of General Surgery, Shanghai Ninth People’s Hospital, Shanghai Jiao Tong University School of Medicine, No. 639, Zhizaoju Road, Huangpu District, Shanghai, People’s Republic of China; 2 Department of Pathology, Shanghai Ninth People’s Hospital, Shanghai Jiao Tong University School of Medicine, No. 639, Zhizaoju Road, Huangpu District, Shanghai, People’s Republic of China

**Keywords:** Gamma-glutamyl transferase, Alanine aminotransferase, Vascular invasion, Hepatocellular carcinoma

## Abstract

**Background:**

The gamma-glutamyl transferase (GGT) to alanine aminotransferase (ALT) ratio has been reported as an effective predictor of the severity of hepatitis and HCC. The purpose of this study was to determine the role of the GGT/ALT ratio in the prediction of vascular invasion and survival outcomes in patients with hepatitis B virus (HBV)-related hepatocellular carcinoma (HCC).

**Methods:**

The risk factors for vascular invasion were determined by univariate/multivariate logistic analysis. The cut-off value of GGT/ALT in predicting vascular invasion was calculated using the receiver operating characteristic (ROC) curve. The prognostic value of GGT/ALT was examined by Cox analysis and Kaplan–Meier curves. Sensitivity analysis, such as subgroup analysis and propensity score matching (PSM), was performed to reduce potential confounding bias.

**Results:**

A high GGT/ALT ratio was identified as an independent risk factor for vascular invasion (*P* = 0.03). The correlation analysis suggested that higher GGT/ALT was associated with more severe tumour burdens, including vascular invasion (*P* < 0.001), tumour volume > 5 cm (*P* < 0.001), poor pathological differentiation (*P* = 0.042), more severe BCLC (*P* < 0.001) and ALBI grade (*P* = 0.007). In the survival analysis, a high GGT/ALT ratio was associated with poor overall survival (OS) (HR: 1.38; 95% CI 1.03, 1.87; *P* < 0.0001) and disease-free survival (DFS) (HR: 1.32; 95% CI 1.03, 1.87; *P* < 0.0001). In the subgroup analysis, similar results were consistently observed across most subgroups. In PSM analysis, GGT/ALT remained independently associated with vascular invasion (OR, 186; 95% CI 1.23, 3.33).

**Conclusion:**

The GGT/ALT ratio was a potential effective factor in the prediction of vascular invasion and prognosis in patients with HBV-related HCC.

## Introduction

In 2018, liver cancer was ranked as the sixth most common cancer and fourth leading cause of cancer-related death in the world, with 841,000 new cases and 782,000 deaths each year [1], among which 85–90% of cases belong to hepatocellular carcinoma (HCC). Hepatitis B virus (HBV) is one of the major risk factors associated with HCC worldwide, especially in China [2].

At present, the treatment of HCC mainly involves chemotherapy, surgical resection and liver transplantation [3]. However, high rates of metastasis and recurrence after surgery severely deteriorate the prognosis; in this process, vascular invasion accounts for the leading cause [4, 5]. Kunutsor et al. [6] demonstrated that intrahepatic vascular invasion was the prevalent cause of postoperative recurrence and cancer-related death in HCC patients. Therefore, accurate and effective assessment of vascular invasion before surgery is strongly needed to guide treatment options.

Currently, preoperative assessment of vascular invasion is mainly performed by computed tomography (CT), while laboratory tests are equally indispensable for adjuvant assessment. Risk factors for HCC vascular invasion include HBV infection, tumour size, multifocal localization, α-fetoprotein (AFP), γ-glutamyl transferase (GGT), alanine transaminase (ALT), etc. [4, 7, 8]. As a major aetiological factor, HBV infection changes the hepatic microenvironment, induces an inflammatory response, and promotes angiogenesis and vascular invasion. Several studies have confirmed the correlation between HBV infection and vascular invasion in HCC [9, 10]. Therefore, it is possible to determine vascular invasion and poor prognosis by assessing HBV severity, in which GGT and ALT have been widely investigated with considerable potential.

A high GGT/ALT ratio was initially found to be prognostically associated with worse condition and treatment response in viral hepatitis [11, 12]. Additional studies indicated that GGT/ALT was a positive predictor of HCC [13, 14]. However, those previous studies emphasized the prediction of hepatitis or HCC more, while the predictive effect of GGT/ALT on vascular invasion in HBV-related HCC is still unknown. Considering the vital role of hepatitis B in vascular invasion of HCC combined with the diagnostic value of GGT/ALT in hepatitis, the aim of our study was to confirm whether GGT/ALT is a risk factor for vascular invasion, cancer severity and outcomes in HBV-related HCC patients.

A total of 558 patients were enrolled in our study. First, potential risk factors for vascular invasion were identified using univariate and multivariate logistic analyses. The cut-off value of GGT/ALT in predicting vascular invasion was calculated using the receiver operating characteristic (ROC) curve. The prognostic value of GGT/ALT was examined by Cox analysis and Kaplan–Meier curves. Finally, subgroup analysis and propensity score matching (PSM) were used to eliminate confounding bias.

## Method

### Patients

Clinical data were collected from 558 patients with HBV-infected HCC who underwent surgical resection at Zhongshan Hospital affiliated with Fudan University (Shanghai, China) between August 1, 2011, and July 31, 2017. The inclusion criteria were as follows: (1) HCC was diagnosed by postoperative pathological examinations; (2) history of HBV infection; (3) patient underwent hepatectomy as initial treatment in Zhongshan Hospital; (4) aged over 18; and (5) no distant metastasis was detected. The exclusion criteria were as follows: (1) patients with alcoholic liver disease, hepatitis C or other primary liver chronic diseases except hepatitis B; (2) patients with other concomitant or previous cancers; and (3) patients with preoperative anticancer treatment such as transarterial chemoembolization (TACE), chemotherapy, radiotherapy, etc.; (4) incomplete clinical and follow-up medical records.

After discharge, patients were followed up regularly every three months for the first three years and every six months thereafter. Serum biomarkers of tumour and hepatitis B were examined periodically, and abdominal contrast-enhanced CT, ultrasound, hepatic arteriogram or invasive examination were performed as needed. Overall survival (OS) was defined as the interval between the date of surgery and death or the last follow-up. Disease-free survival (DFS) was defined as the time interval between the date of surgery and the date of confirmed HCC recurrence or the date of last follow-up. The last follow-up ended on July 31, 2020. The study was approved by the Ethical Committees of Zhongshan Hospital of Fudan University.

### Data collection

The following clinical data of HBV-infected HCC patients were collected in our study: (1) demographic data, including age and sex; (2) preoperative laboratory examination, including GGT, ALT, albumin, AFP and total bilirubin (TB); (3) tumour information evaluated by imaging tests, pathological examinations and scoring systems, including vascular invasion, tumour size, tumour multifocality, tumour capsule, pathological differentiation, cirrhosis, Barcelona Clinic Liver Cancer (BCLC) classification, and albumin-bilirubin (ALBI) grade; and (4) postoperative follow-up data including OS and DFS.

### Definition

Laboratory examination data were manipulated by the laboratory Department of Zhongshan Hospital. HBV infection was defined as a positive test for hepatitis B virus surface antigen (HBsAg). The normal ranges of laboratory tests included ALT ≤ 56 U/L, GGT ≤ 50 U/L, albumin ≤ 40 g/L, TB ≤ 20 μmol/L, and AFP ≤ 400 ng/mL. Tumour size, tumour number, tumour capsule, pathological differentiation, cirrhosis and vascular invasion were confirmed by CT imaging combined with postoperative pathological examination. Each specimen was reviewed independently by two liver pathologists. Microvascular invasion (MVI) is defined as microscopically confirmed tumour clusters in the vascular cavity [4]. The assessment of BCLC classification was performed as described previously [15]. Pathological differentiation was conducted using the Edmondson grading system [16]. ALBI grade was employed as a powerful tool for survival analysis in HCC patients, with a specific calculation process as described previously [17].

### Statistical analysis

The data analysis was performed by R (R Statistical Software, version 4.0.2). The optimal GGT/ALT cut-off value of the receiver operating characteristic (ROC) curve was identified by calculating the maximum value of the Youden index. Categorical variables were assessed using Pearson chi-square test or Fisher's exact test as appropriate. A logistic regression model was used for univariate and multivariate analyses. Cumulative survival rates were calculated by Kaplan–Meier analysis and compared with the log-rank method. The Cox method was used for univariate and multivariate survival analysis. Subgroup analysis was conducted to compare the heterogeneity of prediction in subgroups. *P* < 0.05 was considered statistically significant.

### Propensity score matching (PSM)

PSM was used to reduce bias. The matching process was applied by the minimum distance scoring method combined with matched 1:1 matching within the high and low GGT/ALT groups. Logistic regression models were used to calculate propensity scores, including age, sex, ALT, albumin, TB, AFP, cirrhosis, tumour size, number of tumours, tumour capsule, pathological differentiation, BCLC classification, GGT and ALBI grade. By using the nearest-neighbour PSM algorithm, 93 patients in the high GGT/ALT group were matched 1:1 with 93 patients in the low GGT/ALT group. After matching, univariate logistic analysis was performed between the high and low GGT/ALT groups to predict vascular invasion.

## Results

### General clinical characteristics

**Fig. 1:**
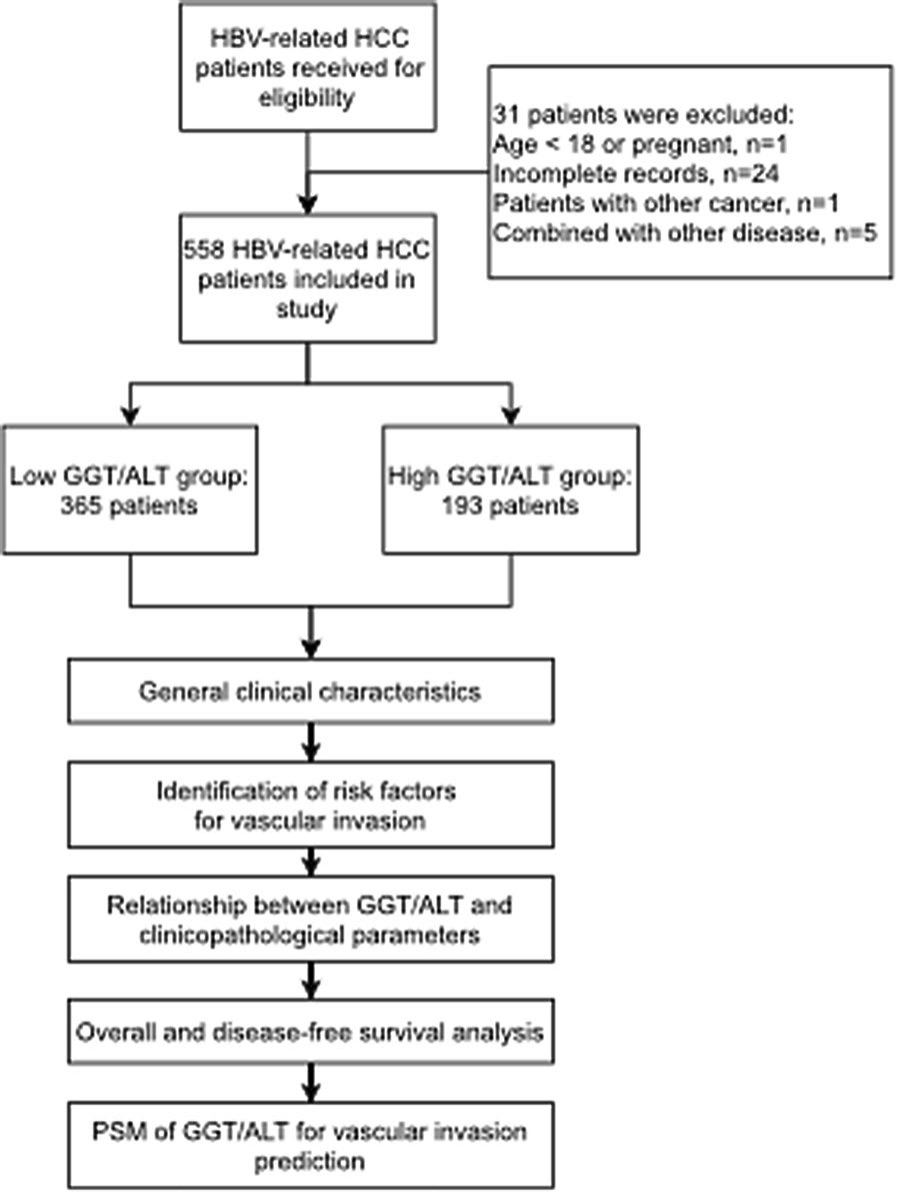
Flowchart of cohort integration

In our study, 558 HCC patients with HBV infection were involved (Fig. 1). All of the patients received tumour resection surgery, and general clinical characteristics are summarized in Table 1. As a consequence of the results, the majority of patients exhibited ALT within the normal range (≤ 56 U/L, 84.41% vs. 15.59%), while the majority of the GGT distributions were comparable between the low (48.75%) and high groups (51.25%). As a sign of poor prognosis, vascular invasion was not combined with most of the patients (69.71% vs. 30.29%). The other principal components included younger than 60 years (75.63% vs. 24.37%), men (84.95% vs. 15.05%), albumin > 40 g/L (51.97% vs. 48.03%), TB < 20 μmol/L (90.14% vs. 9.86%), AFP < 400 ng/mL (72.04% vs. 27.96%), cirrhosis (79.21% vs. 20.79%), tumour size ≤ 5 cm (64.52% vs. 35.48%), solitary tumours (83.87% vs. 16.13%), tumour capsule absence (63.44% vs. 36.56%), and moderate/poor differentiation (67.03% vs. 32.97%). In the BCLC classification, grades A, B, and C accounted for 61.65%, 31.54% and 6.81%, respectively. In ALBI grade, grades A and B accounted for 71.51% and 28.50%, respectively. This finding indicated a significant probability of malignant HBV-associated HCC.

**Table 1:** Distribution of clinical variables among total, high and low GGT/ALT groups

Variables	Total (n = 558)	Low (n = 365)	High (n = 193)	*P*
Age, n (%)				1
≤ 60	422 (75.63)	291 (75.58)	131 (75.72)	
> 60	136 (24.37)	94 (24.42)	42 (24.28)	
Sex, n (%)				0.003
Female	84 (15.05)	70 (18.18)	14 (8.09)	
Male	474 (84.95)	315 (81.82)	159 (91.91)	
ALT, n(%)				< 0.001
≤ 56	471 (84.41)	299 (77.66)	172 (99.42)	
> 56	87 (15.59)	86 (22.34)	1 (0.58)	
Albumin, n (%)				0.057
≤ 40	268 (48.03)	211 (54.81)	79 (45.66)	
> 40	290 (51.97)	174 (45.19)	94 (54.34)	
TB, n (%)				0.657
≤ 20	503 (90.14)	349 (90.65)	154 (89.02)	
> 20	55 (9.86)	36 (9.35)	19 (10.98)	
AFP, n (%)				0.399
≤ 400	402 (72.04)	282 (73.25)	120 (69.36)	
> 400	156 (27.96)	103 (26.75)	53 (30.64)	
Cirrhosis, n (%)				0.904
No	116 (20.79)	79 (20.52)	37 (21.39)	
Yes	442 (79.21)	306 (79.48)	136 (78.61)	
Size, n (%)				< 0.001
≤ 5	360 (64.52)	272 (70.75)	88 (50.87)	
> 5	198 (35.48)	113 (29.35)	85 (49.13)	
Number, n (%)				0.518
Solitary	468 (83.87)	326 (84.68)	142 (82.08)	
Multiple	90 (16.13)	59 (15.32)	31 (17.92)	
Vascular invasion, n (%)				< 0.001
No	389 (69.71)	286 (74.29)	103 (59.54)	
Yes	169 (30.29)	99 (25.71)	70 (40.46)	
Tumor capsule, n (%)				0.962
Absence	354 (63.44)	245 (63.64)	109 (63.01)	
Presence	204 (36.56)	140 (36.37)	64 (36.99)	
Differential, n (%)				0.042
Good	184 (32.97)	269 (69.87)	105 (60.69)	
Moderate/poor	374 (67.03)	116 (30.13)	68 (39.31)	
BCLC, n (%)				< 0.001
A	344 (61.65)	260 (67.53)	84 (48.55)	
B	176 (31.54)	108 (28.05)	68 (39.30)	
C	38 (6.81)	17 (4.42)	21 (12.14)	
GGT, n (%)				< 0.001
≤ 50	272 (48.75)	261 (67.79)	11 (6.36)	
> 50	286 (51.25)	124 (32.21)	162 (93.64)	
ALBI Grade, n (%)				0.007
A	399 (71.51)	289 (75.06)	110 (63.58)	
B	159 (28.50)	96 (24.90)	63 (36.42)	

ALT, Alanine aminotransferase; TB, Total Bilirubin; AFP, α-fetoprotein; BCLC, Barcelona Clinic Liver Cancer staging; GGT, γ-Glutamyl Transpeptidase; ALBI grade, Albumin-Bilirubin Grade

### Identification of risk factors for vascular invasion

Logistic univariate analysis and multivariate analysis were conducted to identify the predictive risk factors for vascular invasion. In univariate analysis, GGT/ALT values were associated positively with vascular invasion (OR: 1.96; 95% CI 1.34, 2.87; *P* = 0.03), the other indicators included age (OR: 0.46, 95% CI 0.29, 0.74; *P* < 0.001), serum AFP (OR: 2.3; 95% CI 1.56, 3.4; *P* < 0.001), tumour size (OR: 2.96; 95% CI 2.04, 4.31; *P* < 0.001), tumour number (OR: 1.68; 95% CI 1.05, 2.68; *P* = 0.032), tumour capsule (OR: 1.79; 95% CI 1.24, 2.59; *P* = 0.002), pathological differentiation (OR: 3.44; 95% CI 2.35, 5.04; *P* < 0.001), BCLC B(OR: 2.95; 95% CI 2.59, 3.002), pathological differentiation (OR: 3.44; 95% CI 2.35, 5.04; *P* < 0.001), BCLC B < 0.0001); ALBI (OR: 2.46, 3.43; P < 0.67).

In further multivariate logistic regression, GGT/ALT (OR: 1.60, 95% CI 1.05, 2.43; *P* = 0.03) was still listed as an independent risk factor for vascular invasion. The other indicators included age (OR: 0.44, 95% CI 0.27, 0.74; *P* = 0.001), serum AFP (OR: 1.63; 95% CI 1.06, 2.51; *P* = 0.026), tumour size (OR: 2.15; 95% CI 1.42, 3.24; *P* < 0.001), tumour capsule (OR: 1.64; 95% CI 1.09, 2.47; *P* = 0.018), pathological differentiation (OR: 2.90; 95% CI 1.93, 4.36; *P* < 0.001), BCLC B (OR: 2.62; 95% CI 2.21, 3.03; *P* < 0.001), BCLC C (OR: 2.76 95% CI 2.33, 3.15; *P* < 0.001), and, as shown in Table 2. The results demonstrated that GGT/ALT was an independent risk factor for predicting vascular invasion in HCC.

**Table 2:** Univariate and multivariate logistic analysis of prognostic factors associated with vascular invasion

Risks	Univariate analysis		Multivariate analysis	
	OR (95% CI)	*P*	OR (95% CI)	*P*
Age, n (%)				
≤ 60	Reference		Reference	
> 60	0.46 (0.29, 0.74)	< 0.001	0.44 (0.27, 0.74)	0.001
Sex, n (%)				
Female	Reference			
Male	1.36 (0.8, 2.32)	0.245		
Albumin, n (%)				
≤ 40	Reference			
> 40	1.26 (0.88, 1.81)	0.208		
TB, n (%)				
> 20	Reference			
≤ 20	0.97 (0.53, 1.77)	0.916		
AFP, n (%)				
≤ 400	Reference		Reference	
> 400	2.3 (1.56, 3.4)	< 0.001	1.63 (1.06, 2.51)	0.026
Cirrhosis, n (%)				
No	Reference			
Yes	1.24 (0.79, 1.97)	0.344		
Size, n (%)				
≤ 5	Reference			
> 5	2.96 (2.04, 4.31)	< 0.001	2.15 (1.42, 3.24)	< 0.001
Number, n (%)				
Solitary	Reference		Reference	
Multiple	1.68 (1.05, 2.68)	0.032	1.44 (0.85, 2.41)	0.175
Tumor capsule, n (%)				
Absence	Reference		Reference	
Presence	1.79 (1.24, 2.59)	0.002	1.64 (1.09, 2.47)	0.018
Differential, n (%)				
Good	Reference		Reference	
Moderate/poor	3.44 (2.35, 5.04)	< 0.001	2.90 (1.93, 4.36)	< 0.001
BCLC, n (%)				
A	Reference			
B	2.95 (2.46, 3.43)	< 0.001	2.62 (2.21, 3.03)	< 0.001
C	3.02 (2.63, 3.72)	< 0.001	2.76 (2.33, 3.15)	< 0.001
ALBI Grade, n (%)				
A	Reference		Reference	
B	1.95 (1.23, 2.67)	0.003	1.44 (0.93, 2.21)	0.1
GGT/ALT				
≤ 2.95	Reference		Reference	
> 2.95	1.96 (1.34, 2.87)	< 0.001	1.60 (1.05, 2.43)	0.030

TB, Total Bilirubin; AFP, α-fetoprotein; BCLC, Barcelona Clinic Liver Cancer staging; ALBI grade, Albumin-Bilirubin Grade; GGT, γ-Glutamyl Transpeptidase; ALT, Alanine aminotransferase

In those variables, pathological features were all positively associated with vascular invasion, which was shown to be linked with more severe tumour situations. The associated factors included tumour size, tumour capsule and pathological differentiation. Three scoring criteria, BCLC and GGT/ALT, were also positively associated with vascular invasion, while ALBI was not related. Interestingly, age was negatively related to vascular invasion, which means that younger patients had a greater proclivity to combine with vascular invasion.

### Correlation between GGT/ALT and HCC related factors

**Fig. 2:**
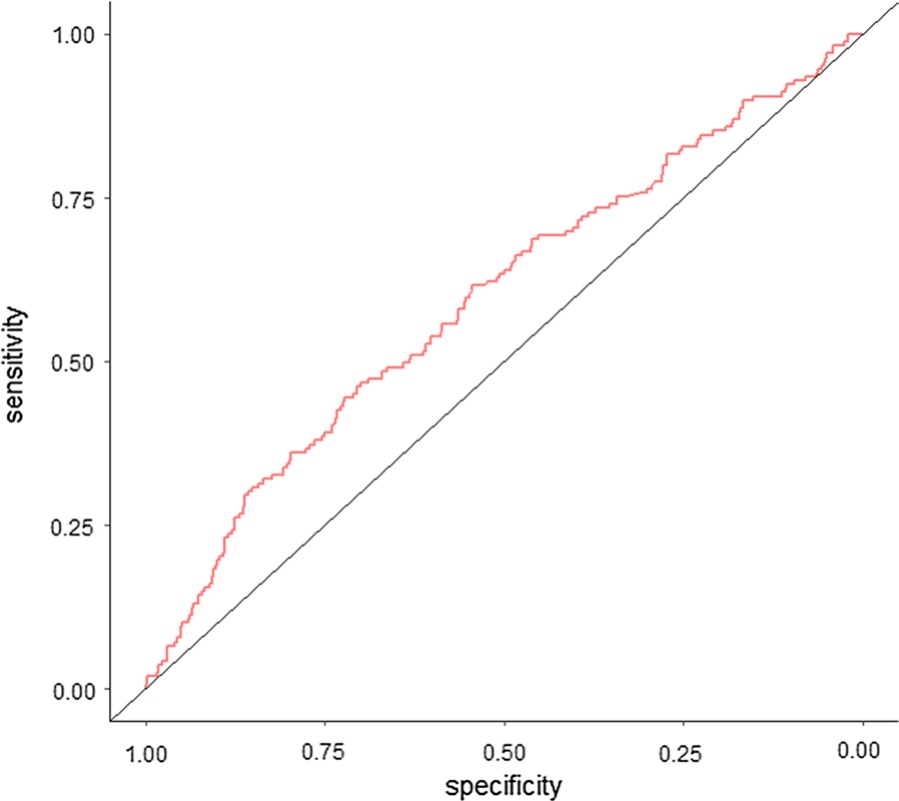
ROC curve of GGT/ALT in predicting vascular invasion for HBV-related HCC patients

The ROC curve of the GGT/ALT ratio in the diagnosis of vascular invasion was plotted and illustrated its predictive value (Fig. 2). The optimal cut-off value of GGT/ALT in the diagnosis of vascular invasion was 2.95 by calculating the Youden index of the ROC curve. Based on this cut-off value of GGT/ALT, the patients were classified into a low group (n = 365) and a high group (n = 193) for further investigations.

The correlations between the GGT/ALT ratio and clinicopathological parameters were examined, as shown in Table 1. The results indicated that the high GGT/ALT group was significantly associated with vascular invasion (*P* < 0.001), and the other related parameters included male sex (*P* = 0.003), ALT < 56 U/L (*P* < 0.001), GGT > 50 U/L (*P* < 0.001), tumour volume > 5 cm (*P* < 0.001), moderate/poor pathological differentiation (*P* = 0.042), more severe BCLC (*P* < 0.001) and ALBI grade (*P* = 0.007). The findings indicated that of the 87 patients with elevated ALT levels, 87 (98.85%) belonged to the low GGT/ALT group, with just 1 patient belonging to the high group. The distribution of GGTs also exhibited comparable characteristics. In a total of 272 patients with GGT ≤ 50 U/L, 261 patients (95.96%) belonged to the low GGT/ALT group. In addition, GGT/ALT also presented a good correlation with tumour severity, which included size, differentiation and classification scores.

**Fig. 3:**
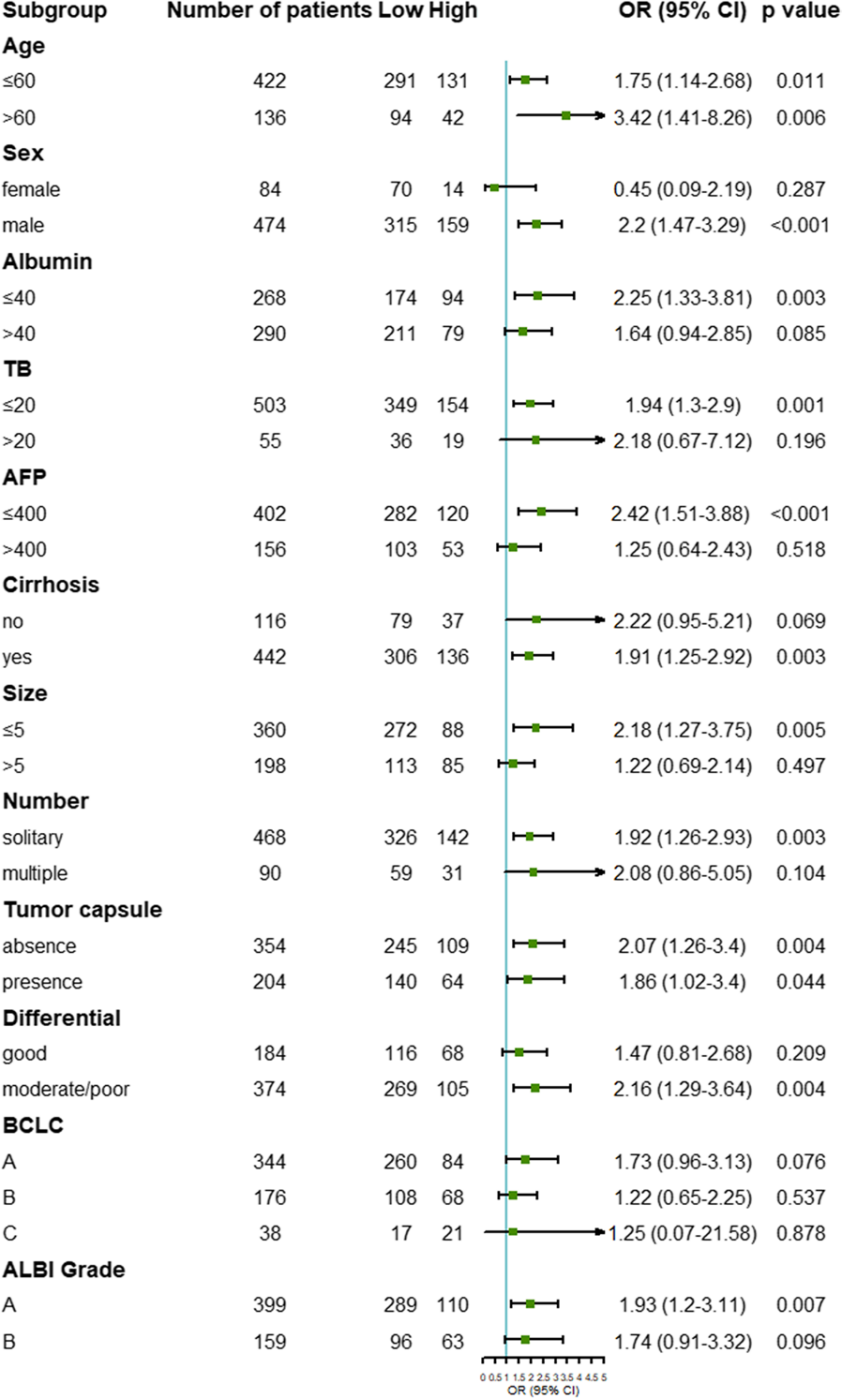
Subgroup analysis for low and high GGT/ALT groups

Next, by using subgroup analysis, we explored the heterogenetic prediction value of GGT/ALT in different subgroups (Fig. 3). The results showed that GGT/ALT had better predictive effects in subgroups of age both ≤ 60 years (OR: 1.75; 95% CI 1.14, 2.68; *P* = 0.011) and > 60 years (OR: 3.42; 95% CI 1.41, 8.26; *P* = 0.006), male (OR: 2.2; 95% CI 1.47, 3.29; *P* < 0.001), albumin ≤ 40 g/L (OR: 2.25; 95% CI 1.33, 3.81; *P* = 0.003), TB ≤ 20 μmol/L (OR: 1.94; 95% CI 1.3, 2.9; *P* = 0.001), AFP ≤ 400 ng/mL (OR: 2.42; 95% CI 1.51, 3.88; *P* < 0.001), cirrhosis (OR: 1.91; 95% CI 1.25, 2.92; *P* = 0.003), tumour size ≤ 5 cm (OR: 2.18; 95% CI 1.27, 3.75; *P* = 0.005), solitary tumour (OR: 1.92; 95% CI 1.26, 2.93; *P* = 0.003), tumour capsule presence (OR: 1.86; 95% CI 1.02, 3.4; *P* = 0.044) or absence (OR: 2.07; 95% CI 1.26, 3.4; *P* = 0.004), moderate/poor pathological differentiation (OR: 2.16; 95% CI 1.29, 3.64; *P* = 0.004), and ALBI stage A (OR: 1.93; 95% CI 1.2, 3.11; *P* = 0.007). In general, the negative predictive value of GGT/ALT was relatively higher in the subgroups above, such as males, older patients, smaller tumour sizes, solitary tumours, etc.

### Cox regression and survival analysis associated with the GGT/ALT ratio

**Fig. 4:**
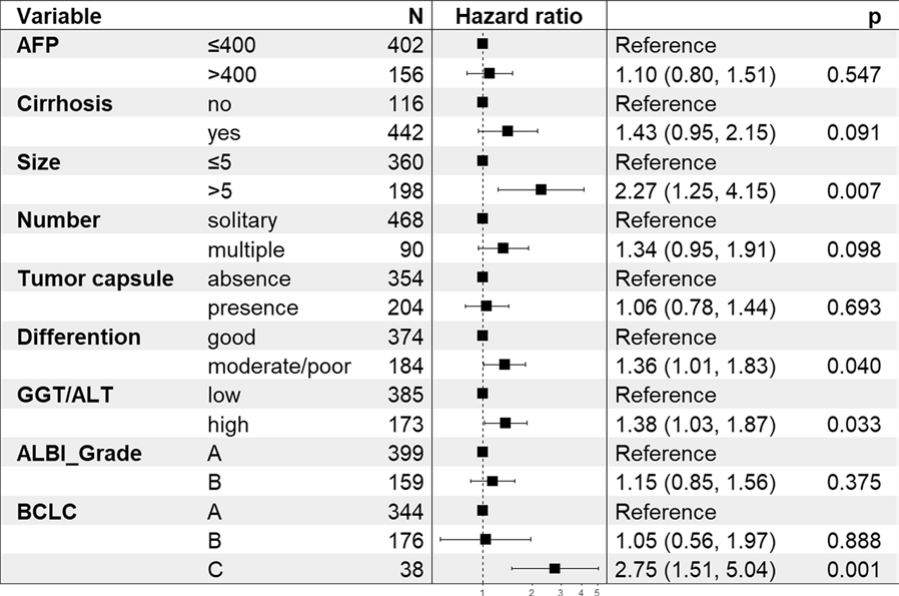
Multivariate Cox regression analysis of overall survival

**Fig. 5:**
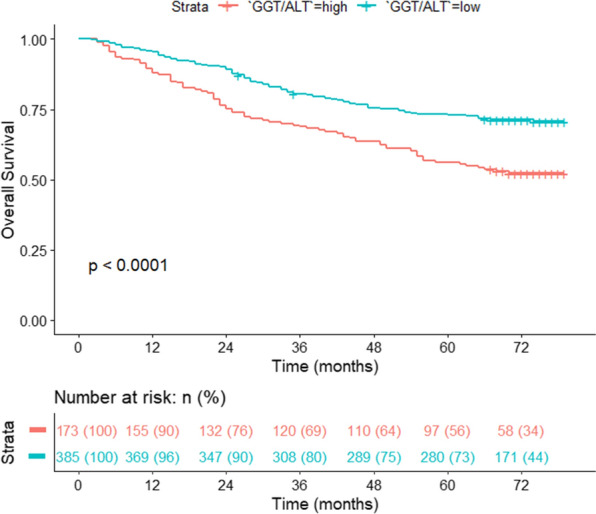
A high ratio of GGT/ALT was correlated with poor overall survival rates

Cox regression and survival analyses were conducted to assess the prognostic factors and confirm the prognostic value of GGT/ALT for OS and DFS. For the analysis of OS, univariate and multivariate Cox regression analyses were performed, and a higher GGT/ALT ratio (OR: 1.38; 95% CI 1.03, 1.87; *P* = 0.033) was identified as a potential prognostic factor for predicting OS in HCC. The other factors included larger tumour size (OR: 2.27; 95% CI 1.25, 4.15; *P* = 0.007), moderate/poor differentiation (OR: 1.36; 95% CI 1.01, 1.83; *P* = 0.040), and BCLC grade C (OR: 2.75; 95% CI 1.51, 5.04; *P* = 0.001), as shown in Fig. 4. Intestinally, ALBI did not demonstrate a significant prognostic value of OS (*P* = 0.375). Subsequently, an OS curve of GGT/ALT was drawn with a total follow-up time of 72 months. The results illustrated that the OS rate of the higher GGT/ALT group decreased significantly (*P* < 0.0001) during the whole follow-up time. The 5-year OS rates were 56.07% in the high group and 72.73% in the low group, as shown in Fig. 5.

**Fig. 6:**
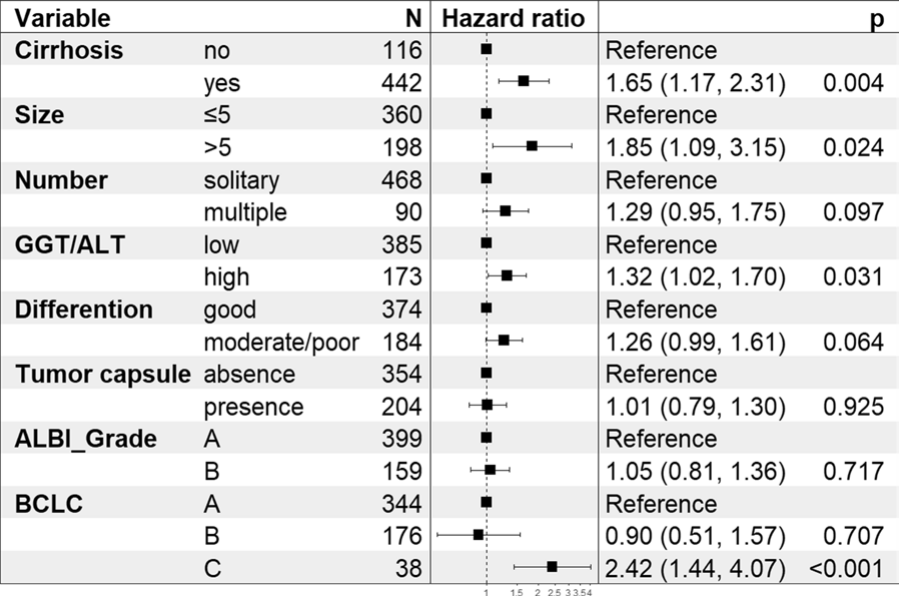
Multivariate Cox regression analysis of disease-free survival

**Fig. 7:**
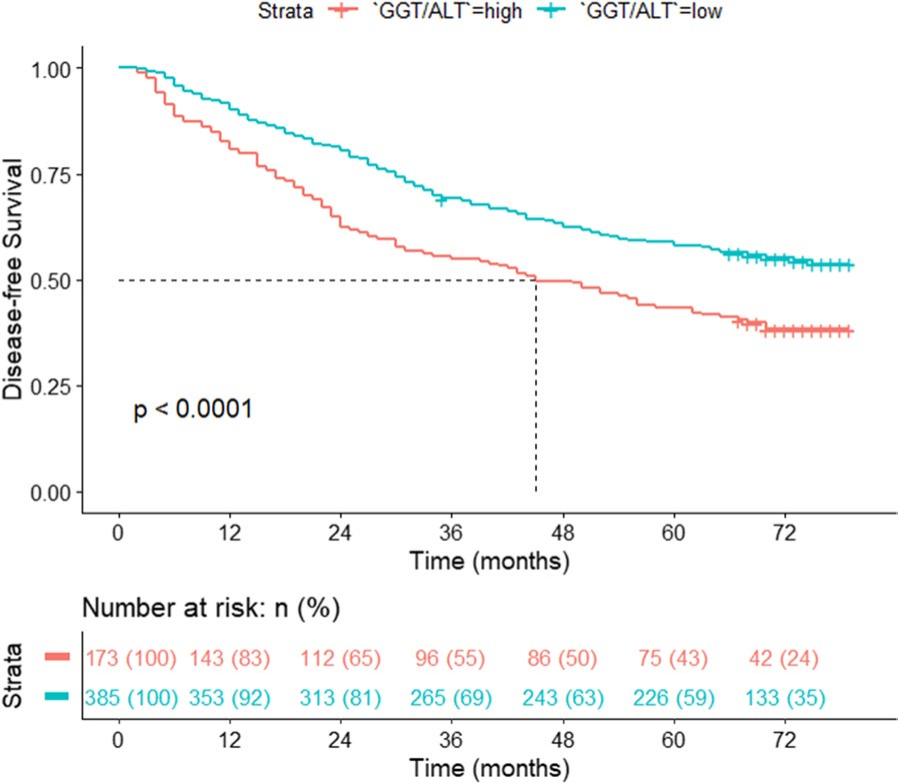
A high ratio of GGT/ALT was correlated with poor disease-free survival rates

For the analysis of DFS, higher GGT/ALT (OR: 1.32; 95% CI 1.02, 1.70; *P* = 0.031) was also outlined as a potential predictor by Cox regression analysis. The other factors included cirrhosis (OR: 1.65; 95% CI 1.17, 2.31; *P* = 0.004), larger tumour size (OR: 1.85; 95% CI 1.09, 3.15; *P* = 0.024) and BCLC grade C (OR: 2.42; 95% CI 1.44, 4.07; *P* < 0.001) (Fig. 6). In this section, ALBI was also omitted from the significant predictors of DFS (*P* = 0.717). The DFS curve of GGT/ALT yielded that the DFS rate of the higher GGT/ALT group was significantly lower, similar to the OS rate (*P* < 0.0001), as shown in Fig. 7. The 5-year DFS rates of the high and low GGT/ALT groups were 43.35% and 58.70%, respectively.

### PSM of GGT/ALT for vascular invasion prediction

To avoid the interference of other clinical parameters, PSM was used to match the data of the high and low GGT/ALT groups. After matching, there were 93 patients in the low and high GGT/ALT groups. In the original data, differences in distributions were characterized in several variables, including sex (SD = 0.302), ALT (SD = 0.727), albumin (SD = 0.184), tumour size (SD = 0.469), tumour differentiation (SD = 0.194), BCLC (SD = 0.424), GGT (SD = 1.648) and ALBI grade (SD = 0.251). After matching, there was no significant difference in the distribution between the two groups except cirrhosis (SD = 0.132), as shown in Table 3.

**Table 3:** Absolute standardized differences before and after propensity score matching

Variables	Before matching		SD	After matching		SD
	Low (n = 365)	High (n = 193)		Low (n = 93)	High (n = 93)	
Age, n (%)			0.003			0.077
≤ 60	291 (75.58)	131 (75.72)		73 (78.49)	70 (75.27)	
> 60	94 (24.42)	42 (24.28)		20 (21.51)	23 (24.73)	
Sex, n (%)			0.302			0.032
Female	70 (18.18)	14 (8.09)		12 (12.90)	13 (13.98)	
Male	315 (81.82)	159 (91.91)		81 (87.10)	80 (86.02)	
ALT, n(%)			0.727			< 0.001
≤ 56	299 (77.66)	172 (99.42)		92 (98.92)	92 (98.92)	
> 56	86 (22.34)	1 (0.58)		1 (1.08)	1 (1.08)	
Albumin, n (%)			0.184			0.086
≤ 40	211 (54.81)	79 (45.66)		49 (52.69)	45 (48.39)	
> 40	174 (45.19)	94 (54.34)		44 (47.31)	48 (51.61)	
TB, n (%)			0.054			0.077
≤ 20	349 (90.65)	154 (89.02)		86 (92.47)	84 (90.32)	
> 20	36 (9.35)	19 (10.98)		7 (7.53)	9 (9.68)	
AFP, n (%)			0.086			0.023
≤ 400	282 (73.25)	120 (69.36)		65 (69.89)	64 (68.82)	
> 400	103 (26.75)	53 (30.64)		28 (30.11)	29 (31.19)	
Cirrhosis, n (%)			0.021			0.132
No	79 (20.52)	37 (21.39)		17 (18.28)	22 (23.66)	
Yes	306 (79.48)	136 (78.61)		76 (81.72)	71 (76.34)	
Size, n (%)			0.469			0.088
≤ 5	272 (70.75)	88 (50.87)		61 (56.99)	49 (52.69)	
> 5	113 (29.35)	85 (49.13)		40 (43.01)	44 (47.31)	
Number, n (%)			0.07			< 0.001
Solitary	326 (84.68)	142 (82.08)		77 (82.80)	77 (82.80)	
Multiple	59 (15.32)	31 (17.92)		16 (17.20)	16 (17.20)	
Tumor capsule, n (%)			0.013			0.091
Absence	245 (63.64)	109 (63.01)		64 (68.82)	60 (64.52)	
Presence	140 (36.37)	64 (36.99)		29 (31.18)	33 (35.48)	
Differential, n (%)			0.194			0.067
Good	269 (69.87)	105 (60.69)		32 (34.41)	35 (37.63)	
Moderate/poor	116 (30.13)	68 (39.31)		61 (65.59)	58 (62.37)	
BCLC, n (%)			0.424			0.092
A	260 (67.53)	84 (48.55)		51 (54.84)	51 (54.84)	
B	108 (28.05)	68 (39.3)		37 (39.78)	35 (37.63)	
C	17 (4.42)	21 (12.14)		5 (5.38)	7 (7.53)	
GGT, n (%)			1.648			< 0.001
≤ 50	261 (67.79)	11 (6.36)		11 (11.83)	11 (11.82)	
> 50	124 (32.21)	162 (93.64)		82 (88.17)	82 (88.17)	
ALBI Grade, n (%)			0.251			0.024
A	289 (75.06)	110 (63.58)		65 (69.89)	66 (70.97)	
B	96 (24.9)	63 (36.42)		28 (30.11)	27 (29.03)	

ALT, Alanine aminotransferase; TB, Total Bilirubin; AFP, α-fetoprotein; BCLC, Barcelona Clinic Liver Cancer staging; GGT, γ-Glutamyl Transpeptidase; ALBI grade, Albumin-Bilirubin Grade

After matching, univariate logistic analysis was performed to evaluate the potential risk factors in the prediction of vascular invasion in HCC. The results showed that GGT/ALT (OR: 1.86; 95% CI 1.23, 3.33; *P* = 0.037) was still correlated with vascular invasion. The other risk factors comprised age (OR: 0.36; 95% CI 0.15, 0.87; *P* = 0.015), AFP (OR: 2.11; 95% CI 1.08, 4.12; *P* = 0.029), tumour size (OR: 2.68; 95% CI 1.42, 5.07; *P* = 0.002), tumour capsule (OR: 2.05; 95% CI 1.09, 3.87; *P* = 0.026), pathological differentiation (OR: 4.71; 95% CI 2.42, 9.17; *P* < 0.001), BCLC grade B (OR: 2.61; 95% CI 1.32, 5.15; *P* = 0.006), and grade C (OR: 2.88; 95% CI 2.18, 3.42; *P* < 0.001), as shown in Table 4. Additionally, the ALBI grade was not significantly associated with vascular invasion after PSM (*P* = 0.485). The results confirmed GGT/ALT as a good predictive value for vascular invasion.

**Table 4:** Univariate logistic regression associated with vascular invasion after PSM

Risks	Univariate analysis	
	OR (95% CI)	*P*
Age, n (%)		
≤ 60	Reference	
> 60	0.36 (0.15, 0.87)	0.015
Sex, n (%)		
Female	Reference	
Male	1.58 (0.6, 4.2)	0.342
Albumin, n (%)		
≤ 40	Reference	
> 40	1.37 (0.74, 2.56)	0.315
TB, n (%)		
≤ 20	Reference	
> 20	1.6 (0.5, 5.12)	0.417
AFP, n (%)		
≤ 400	Reference	
> 400	2.11 (1.08, 4.12)	0.029
Cirrhosis, n (%)		
No	Reference	
Yes	0.93 (0.42, 2.08)	0.864
Size, n (%)		
≤ 5	Reference	
> 5	2.68 (1.42, 5.07)	0.002
Number, n (%)		
Solitary	Reference	
Multiple	1.24 (0.6, 2.55)	0.567
Tumor capsule, n (%)		
Absence	Reference	
Presence	2.05 (1.09, 3.87)	0.026
Differential, n (%)		
Good	Reference	
Moderate/poor	4.71 (2.42, 9.17)	< 0.001
BCLC, n (%)		
A	Reference	
B	2.61 (1.32, 5.15)	0.006
C	2.88 (2.18, 3.42)	< 0.001
ALBI Grade, n (%)		
A	Reference	
B	1.27 (0.65, 2.46)	0.485
GGT/ALT		
≤ 2.95	Reference	
> 2.95	1.86 (1.23, 3.33)	0.037

TB, Total Bilirubin; AFP, α-fetoprotein; BCLC, Barcelona Clinic Liver Cancer staging; ALBI grade, Albumin-Bilirubin Grade; GGT, γ-Glutamyl Transpeptidase; ALT, Alanine aminotransferase

## Discussion

Our study demonstrated the GGT/ALT ratio as an independent predictive biomarker for vascular invasion in HBV-related HCC. After grouping patients with a cut-off value of 2.95, the high GGT/ALT group showed positive predictive value for vascular invasion, higher tumour severity, and lower DFS and OS in HCC patients. Further sensitivity analysis, including subgroup analysis and PSM, was performed and demonstrated GGT/ALT as an independent predictor for vascular invasion in HCC patients.

Vascular invasion is one of the major factors leading to poor prognosis of HCC, thus severely influencing the treatment effect of resection surgery [18]. Therefore, timely and accurate evaluations are pivotal for guiding therapeutic approaches and improving survival. As a main aetiology of HCC, HBV infection has been proven to be an important causative agent of vascular invasion in HCC patients.

HBV infection leads to multiple pathophysiological alterations, including DNA oxidative damage, liver cell necrosis, inflammatory responses, cytokine synthesis and release, fibrosis and tumour tumorigenesis [19]. In this process, HBV X protein (HBx) has been found to be related to MVI development involved in postoperative recurrence [20, 21]. Yang et al. [10] found that HBV-positive patients were more prone to develop vascular invasion in HCC. In further clinical studies, Lei et al. [9] discovered that a preoperative HBV DNA load larger than 104 IU/mL was an independent risk factor for vascular invasion in HBV-related HCC. Wei et al. [22] further summarized that both infection and active replication of HBV were associated with inflammatory injuries, the occurrence of vascular invasion and cancer metastasis in liver cancer. In addition to aggravating the progression and vascular invasion of HCC, Sarbarzeh et al. [23] also discovered that hepatitis may result in severe psychological issues. Considering the increased risk of vascular invasion associated with HBV infection, it is critical to predict the vascular invasion in HBV-related HCC.

Among the examinations, GGT and ALT are adopted broadly in the evaluation of liver pathology [24–28]. GGT can mediate the production of ROS, promote cell growth and proliferation [29] and is often elevated in liver diseases such as hepatitis, cancer and vascular invasion [25, 26, 30].ALT is also known as a marker of liver dysfunction and inflammation, which was found to be associated with recurrence and poor survival of HBV-related HCC [31, 32]. However, the changes in different biomarkers were not equally accompanied by fluctuations in inflammatory responses. In HBV-related HCC patients, the prediction value of GGT for vascular invasion might be interfered by chronic inflammation and poor liver reserves associated with HBV infection. Therefore, the combination of GGT/ALT might exerts better predictive accuracy than using GGT alone [33].

Ebiling et al. [11] disclosed that a higher GGT/ALT ratio could predict a worse prognosis in chronic hepatitis C. The research of Tarantino et al. [12] further supported this idea by the discoveries that a lower GGT/ALT ratio is an independent predictor of antiviral therapy response. Consequently, GGT/ALT could reflect the hepatitis severity such as prognosis and therapy responses, which made it possible to predict hepatitis-induced HCC. Additionally, previous research also indicated that the high-level GGT/ALT ratio was associated more with tumour burden rather than inflammatory hepatitis parameters [13].

Several studies have evaluated the predictive value of GGT/ALT in liver cancer. Yang et al. [10] observed that hepatitis B patients with a higher GGT/ALT ratio have an increased risk of developing primary hepatic malignancy. Additionally, it was documented in HBV-related HCC patients with Child–Pugh A class that an elevated GGT/ALT ratio was associated with more severe tumour burden, including tumour size, vascular invasion, tumour capsule, and shortened survival time [13]. However, the study above generally focused on the predictive value of GGT/ALT for tumour severity, in which vascular invasion is just a subpart of the symptoms. Therefore, it is still debatable whether GGT/ALT remains a good predictor of vascular invasion in HCC when evaluated independently. Therefore, we focused for the first time on the risk factors for vascular invasion and investigated the independent predictive value of GGT/ALT. Combining the pathological role of HBV infection in vascular invasion and thus in HCC progression and the predictive value of GGT/ALT in both hepatitis B and HCC, we conducted this study to confirm whether GGT/ALT is an independent predictive factor of vascular invasion and outcomes in HBV-infected HCC.

As mentioned above, GGT/ALT has the ability to predict vascular invasion in HCC. However, the different distributions of characteristics among HCC patients increased the heterogeneity and might lead to false positive results. Therefore, sensitivity analysis, including subgroup analysis and PSM, was conducted. In the subgroup analysis, the predictive value of GGT/ALT was examined effectively in most subgroups. The insignificant predictive value in other subgroups might be attributed to the relatively small number of cases, and this could be confirmed through a larger database in the future. Moreover, PSM was employed to balance the distributions of variables between the high and low GGT/ALT groups. After matching, GGT/ALT was still listed as an independent risk factor for vascular invasion, which proved the predictive value of the ratio alone.

Currently, there is no authoritative explanation for the predictive value of GGT/ALT. One possible hypothesis is proposed as follows: GGT is affected by both inflammation and tumours, while ALT is relatively more responsive to hepatic inflammation and liver functions. Although hepatitis in HCC patients has often been controlled before resection, GGT and ALT might still be interfered with by residual HBV infection or surgical stimulus. Therefore, the ratio of GGT/ALT could reflect the stages of primary tumour progression more precisely by minimizing the interference of inflammation. In addition, decreased ALT also indicated an increased mortality rate in people aged over 60 years because of the depletion of liver function reserve, which could explain the positive correlation between the GGT/ALT ratio and worse prognosis [34]. Another potential explanation is that GGT/ALT ratio was also confirmed as an independent predictor of antiviral therapy response and prognosis in chronic hepatitis, which was associated with an increased risk of tumorigenesis and vascular invasion [11, 12].

In addition to HBV, the etiology of HCC also includes hepatitis C virus (HCV), food contaminated with aflatoxin, heavy drinking, obesity, smoking, type 2 diabetes, etc. [1]. Considering the degree of inflammation and vascular invasion may vary among the different etiologies, the predictive value of GGT/ALT would also be investigated respectively in the future.

In clinical practice, we often get perplexed by the fact that different patients may have totally dissimilar vascular invasions and related prognosis. However, current assessment techniques, such as CT scans and tumour markers, are insufficient to detect high risk patients. BCLC classification has also exhibited good predictive value in HCC. However, BCLC is relatively difficult to address, which limits its clinical use. As a powerful complement to imaging examination, scoring systems and other screening methods [35], our study provided a simple and feasible monitoring tool to assess vascular invasion, guide the surgical approach and predict the outcomes of HCC, which makes it easier to manage patients. With more indicators identified in the future, the evaluation model for HCC vascular invasion will be continuously refined with the input of further experimental data.

In addition to GGT/ALT, there are many other biomarkers of vascular invasion, such as Vascular-endothelial cadherin (CDH5), Angiopoietin-2 (ANGPT2), ETS-related gene (ERG), etc. [36]. Relatively, serum GGT/ALT is easier to obtain and measure in clinical use. Therefore, it could be used in the preoperative evaluation of vascular invasion, postoperative follow-up, recurrence surveillance, and prognostic assessment. As contrast, tissue biomarkers might have better potential diagnostic performance and long-term prognostic value. However, potential bias also existed according to distinct parts of tissue and different pathologists. The specimen collection during surgery also restricts its preoperative assessment. Therefore, it does require long-term and extensive research.

This study still had several limitations. First, this study only involves a single centre. More cases from different centres and regions are required in the future. Second, this study is a retrospective study, which may lead to selection bias. This may be further verified by large-scale, randomized, controlled trials in the future.

## Conclusion

Our study proposed relatively reliable evidence in proving the prognostic value of GGT/ALT. As a cheap and convenient biomarker, GGT/ALT is an independent predictor of vascular invasion and outcome in HBV-related HCC patients undergoing resection surgery. As a screening tool, GGT/ALT could help to optimize the treatment strategies for HCC patients and improve survival after surgery.

## Data Availability

The datasets generated and analysed during the current study are not publicly available, as the data are being used in the next study but are available from the corresponding author upon reasonable request.

## Abbreviations

HCC: Hepatocellular carcinoma
HBV: Hepatitis B virus
CT: Computed Tomography
AFP: α-Fetoprotein
GGT: γ-Glutamyl transferase
ALT: Alanine transaminase
ROS: Reactive oxygen species
TACE: Transarterial chemoembolization
OS: Overall survival
DFS: Disease-free survival
TB: Total bilirubin
BCLC: Barcelona Clinic Liver Cancer
ALBI: Albumin-Bilirubin
HBsAg: Hepatitis B virus surface antigen
ROC: Receiver operating characteristic
PSM: Propensity score matching
MVI: Microvascular invasion
HCV: Hepatitis C virus
CDH5: Vascular-endothelial cadherin
ANGPT2: Angiopoietin-2
ERG: ETS-related gene
